# Spanish Version of the Attitude Towards Euthanasia Scale

**DOI:** 10.3390/ijerph17113855

**Published:** 2020-05-29

**Authors:** Elia Fernández-Martínez, Juan José Fernández-Muñoz, Cristina Romero-Blanco, María Laura Parra-Fernández, María Dolores Onieva-Zafra

**Affiliations:** 1 Department of Nursing, University of Huelva, 21071 Huelva, Spain; elia.fernandez@denf.uhu.es; 2 Department of Psychology, Universidad Rey Juan Carlos, 28922 Alcorcón, Madrid, Spain; juanjose.fernandez@urjc.es; 3 Department of Nursing, Physiotherapy and Occupational Therapy, University of Castilla-La-Mancha, Ciudad Real, 13071 Ciudad Real, Spain; Cristina.romero@uclm.es (C.R.-B.); MariaDolores.Onieva@uclm.es (M.D.O.-Z.)

**Keywords:** attitude, end-of-life care, euthanasia, psychometric properties

## Abstract

Euthanasia is undoubtedly the protagonist of many of the debates around the end of life both among health staff and in the general population. Considering that nurses provide care for terminally ill patients and support families and patients in their final days, it is essential to know their attitudes towards euthanasia. The aims of the study were to adapt and validate the Attitude Towards Euthanasia scale to a Spanish context, to test the dimensionality and to estimate the reliability of the scale. A cross-sectional study was conducted with a non-probabilistic sample of Spanish health-workers of 201 in a University Hospital in Ciudad Real. A self-reported socio-demographic questionnaire and the Euthanasia Attitude Scale were used for data collection. The psychometric properties of the scale were assessed, including reliability and validity using an exploratory and confirmatory factor analysis. Cronbach’s alpha of the Attitude Towards Euthanasia scale was α = 0.827 and McDonald’s Omega = 0.903. The range of items of homogeneity was from 0.205 to 0.685. For the different exploratory factor analyses carried out, the Bartlett’s test of sphericity was *p* < 0.001 and the sample index value of Kaiser-Meyer-Olkin was over 0.802. in all cases. We present the factorial weights for three models: The first one assumes a unidimensional solution, the second model was composed by three factors and the third model was composed by four factors. In the confirmatory factor analysis, the three models presented an acceptable fit index. The Attitude Towards Euthanasia scale adaptation to a sample of Spanish health workers has shown, with some limitations, appropriate psychometric properties. There have been several differences between the original factorial solution. It would be necessary to replicate the study to reinforce the findings about the number of factors of the scale.

## 1. Introduction

The term euthanasia itself comes etymologically from Greek, and simply means “good death,” “peaceful death,” or “death without suffering” [1]. However, the different classifications as active or passive, direct or indirect, and even the different related concepts such as assisted suicide, orthotasia, distanasia, etc. make it quite confusing. We can differentiate direct euthanasia (the process of bringing forward the death of a person who has an incurable disease) from indirect euthanasia, although some authors prefer not to quote this term as they consider that there is no euthanasia if there is no intention to cause death [2]. Within direct euthanasia we can distinguish two types, active euthanasia, which achieves the death of the patient through the use of drugs that are lethal, and passive euthanasia, which consists of achieving the death of the patient through the suspension of both medical treatment and food by any means [3].

In Spain, as in other countries, the term euthanasia is surrounded by media controversy, colored by social, cultural and, of course, religious factors [4]. Today, there are debates about the legalization of euthanasia around the world, accompanied by a wide variety of political and ideological groups with very diverse voices, ranging from extensive pro-euthanasia campaigns to strong opponents of legalizing euthanasia. Currently in this country, despite various attempts to regulate the issue of euthanasia, it is still criminalized by law. In the last legislature, the government promoted a proposed law to regulate euthanasia and thus become the fourth country in Europe to legalize euthanasia, after the Netherlands, Luxembourg and Belgium. However, this law was not even voted on in the Spanish Parliament because it was postponed due to the imminent call for general elections. What does exist in Spain is a Law of Patient Autonomy and Rights and Obligations, according to which the patient has the right to decide freely, after receiving adequate information, among the available clinical options”, as well as to “refuse treatment, except in the cases determined by law [4].

The opinions and/or attitudes held by different health professionals or even patients and families themselves have been the subject of research in different countries around the world regardless of whether or not euthanasia was legalized in the country. The fact that it is so important to know the attitude of the professionals is probably linked to the fact that they will be in charge of assessing and carrying out the patient’s request, without forgetting on the other hand, that every health professional has the right to conscientious objection. This is something that, in Spain, is very widespread in healthcare practice, for example in voluntary abortion where in most public hospitals it is not performed due to conscientious objection and private centers must be used in concert with the public health system where they are exclusively dedicated to performing them [5].

Regarding the different factors that have been observed and that have a direct influence on the opinion/attitudes that health professionals have towards euthanasia, of special relevance we find religion as one of the most predictive factors and in negative correlation with support for euthanasia [6,7,8,9,10] Gender, educational level, marital status and number of children are some of the factors that other researchers have studied [11,12,13].

Few studies have explored the attitude of health professionals in Spain towards this issue, and in most of these attempts, a tool validated and used in other countries has never been used. Recently Onieva et al. have validated the Euthanasia Attitude Scale (EAS) tool in a sample of university nursing students with good psychometric properties [4]. However, we may face different attitudes depending on whether we are talking about active or passive euthanasia, just as there are certain situations where people may be more for or against euthanasia. This can happen in cases where there is no possibility of recovery for that person, and ultimately when the terminally ill person is in severe pain, which is the focus of most popular discussions today. Finally, the autonomy of the patient in relation to the authority of the physician would be another great obstacle to resolve when we analyze the attitude towards euthanasia. Considering all these factors, Wassermann et al. developed the Attitude Towards Euthanasia (ATE) scale, which takes into account the different conceptual dimensions to be dealt with in each of the items [14]. However, according to the authors, no dimension should be discussed in isolation from the other dimensions [15].

The aim of this study was to adapt and to validate the ATE scale to a Spanish context, to test the dimensionality of the scale by means of confirmatory factor analysis (CFA), and to estimate the reliability of the scale.

## 2. Materials and Methods

### 2.1. Design, Setting and Participants

The development of the translated and adapted Spanish version of the scale followed the usual recommendations, according to the following two steps: (1) cross-cultural translation in which two translations of the original version were made in the corresponding language of the target population by two translators whose mother tongue was Spanish. A back-translation of the same was then performed by professionals whose mother tongue was English and (2) a psychometric assessment of the validity and reliability of the Spanish version of the ATE. Before the performance of the second part of the process, a research panel, consisting of experienced nurses, reviewed the translated version in order to obtain a semantic and technical equivalence. After this, a pilot study of the final draft was performed with five Spanish-speaking nurses who were associated professors of the nursing faculty in order to assess the comprehension of the questionnaire.

Participants were 201 health workers from a Spanish University Hospital; A prior sample size calculation was not performed, rather the sample size was based on the entire population of health professional at the Hospital. The exclusion criteria consisted of those health workers who were not willing to participate. For ethical reasons, we could not inquire about the causes leading them to reach this decision. The participants received no financial incentive.

The study was approved by the Ethics Committee of the University Hospital of Castilla-La Mancha, according to the ethical guidelines established by the Helsinki Declaration in 2008 (Code C- 153). Written consents were obtained from the participants.

### 2.2. Instruments

Demographic information: Participants’ self-reported sociodemographic characteristics, including age, gender, marital status, educational level and nuclear family and other variables related to practice or no a religion.

The ATE scale is a ten-item instrument directed towards assessing attitudes towards euthanasia developed by Wasserman, Claire and Ritchie in 2005 [14,15]. The Cronbach´s Alpha for the original scale was 0.87. The answer´s scale is a Likert response from 1 to 5 where: (1) strongly disagree, (2) disagree, (3) undecided, (4) agree, and (5) strongly agree. Two items were reversed coded to check against response bias form the participants. Examples of items are: “if a patient in severe pain requests it, a doctor should remove life support and allow that patient to die” or “it is okay for a doctor to administer enough medicine to a suffering patient to end that patient’s life if the doctor thinks that the patient´s pain is too severe”. The original factorial structure was composed by four dimension: severe pain [1,3,9], no recovery [4,6], patient request [8,10] and doctor´s authority [4,5,7]. Several items had cross-loaded with several dimension. The Persian version of the ATE scale showed to have high internal consistency reliability, with Cronbach an at 0.90 and a 2-factor structure representing voluntary and non-voluntary euthanasia [6].

### 2.3. Data Analysis

Statistical analyses were conducted with SPSS 24.0, AMOS 19.0. Three EFA factor-solutions were explored in the study: (i) the one-factor solution because some authors have interpreted ‘Euthanasia Attitude’ is unidimensional, (ii) a three-factor solution based on Eigenvalues >1, and (iii) a four-factor solution as suggested by the original authors. For the Exploratory Factor Analysis (EFA) a Varimax rotation method was conducted; Kaiser- Meyer-Olkin (KMO) and Bartlett’ss test of sphericity were assessed in order to check the adequacy of the data; Cut off points less than 0.30 were excluded of rotated component matrix [16]. For the CFA, the factorial solution with one, three and four factors were used in order to assess the fit of each model; the indices used for that were: Akaike Information Criterion (AIC), Bayesian Information Criterion (BIC) X2, adjusted goodness of fit index (AGFI): values over 0.90 imply an acceptable model; Comparative fit index (CFI), normed fit index (NFI) and Tucker-Lewis coefficient (TLI). In all cases the range of values should be between 0 and 1 and the reference value is over 0.90 [17]. Standardized room mean square residual (SRMR) and root mean square error of approximation (RSMEA) were also checked for the overall fit. In this sense, for both indices lower values involve better fit with a reference value of 0.06 [18,19]. Finally, in order to assess reliability scale several indices was applies such as Cronbach´s Alpha [20], Omega and Coefficient H [17,18,21].

## 3. Results

Almost three quarters (70.6%) of the participants were female. The mean age of the sample was 40.70 (SD = 11.01) with an age range of 18–68. 39.8% were single, 50.7% married, 8.0% divorced and 1.5% widowed. 23.9% were nursing assistant, 65.6% nurses and 10.5% were doctors. The 90.5% work a full time and 9.5% half a day. 14.9% of thsese workers had contact with patient kids, 56.2% with adults and 28.9% with elderly patients and furthermore, the 57.7% profess a religion.

Table 1 shows the descriptive analysis, item homogeneity, and α if item is deleted for the ten items of ATE scale. In this sense, the mean range was between 1.57 (SD = 1.07) for the item 7 to 0.247 (SD = 1.18) for the item 8. Item homogeneity ranged between 0.205 to 0.685. The lowest values for the items of homogeneity were in the ATE 6 (0.205) and ATE 9 (0.305). Both items were phrased negatively and had to be reverse coded.

### Exploratory and Confirmatory Factor Analysis

In order to assess Waserman et al.’s solution we have carried out several exploratory factor analyses (EFA). In all cases the Barlett´s test of sphericity was *p* < 0.001 and the sample index values of KMO was over 0.802. Table 2 presents the factorial loadings for three models. The first one assumes a unidimensional solution. In this case, the factorial weights were between 0.365 (item 6) and 0.790 (item 2). Cronbach’s alpha of the ATE scale was α = 0.827, Omega Ω = 0.903 and H = 0.893. The second model was composed by three factors: 2, 4, 5 and 7 for the first one; 8 and 10 for the second one and 1 and 3 for the third one; in this case, the factorial weights were between 0.704 (item 7) and 0.898 (item 8); Finally, M3 was composed of four factors: 2, 4, 5 and 7 for the first one 8 and 10 for the second one 1 and 3 for the third one; and finally 6 and 9 for the fourth factor; in this model, the factorial weights were between 0.711 (item 2) and 0.897 (item 10); According to Waserman et al., in M2 and M3 the first factor could be the dimension “Doctor´s authority” (α = 0.822, Ω = 0.774 and H = 0.853); the second factor could be named “Patients request” (α = 0.798, Ω = 0.646 and H = 0.869); the third factor would be “severe pain” (α = 0.767, Ω = 0.629 and H = 0.828). At the M3 the fourth factor could be called “no recovery” (α = 0.450, Ω = 0.653 and H = 0.769) although it is not similar according to the items to the original dimension. This factor is composed by “even if a doctor does not think that a patient will recover, it would be wrong for the doctor to end the life of a patient” and “even if a doctor knows that a patient is in severe, uncontrollable pain, it would be wrong for the doctor to end the life of that patient.

CFA was carried out on the data in order to replicate the factor solution of Waserman et al. and also a unidimensional solution. In this sense, the three models had an acceptable fit (see Table 3). The M_3_ could be the considered the model with the best model fit; in this case, AGFI, CFI, NFI, TLI had scores over of the reference value 0.90, RMR were less than 0.06 and RMSEA less than 0.08. The M_1_, M_2_ and M_4_ did not have a good model fit. Therefore, the original model, M4, did not provide an acceptable fit to the data. Among the models with acceptable fits, M1–M3, M3 is selected because of the interpretability of the factors.

Moreover, Table 4 shows the three models applied in this study (with item distributions) and their comparison with the Waserman et al. model.

## 4. Discussion

The ATE scale adaptation to a sample of Spanish health workers has shown, with some limitations, appropriate psychometric properties. There have been several differences between the original factorial solution and this study.

On the one hand, regarding the internal consistency the findings were similar to Waserman, Claire and Ritchey [14] and the overall reliability and homogeneity indices were as high as the original study. The index of internal consistency for the fourth factor of the third model was poor. In M3 the fourth factor is named as a “no recovery” because this dimension has not appeared in the M2. This dimension in our model was composed by two items, 6 and 9. In the original test by Waserman et al. this dimension is composed of items 4 and 6. Item 9, according to the original scales, should have been in the severe pain factor. In this sense, it could be more reasonable to be placed in the dimension related with severe pain that in “no recovery” but at the same time Wasserman et al. suggested to discuss all the dimensions together and not in isolation, a patient with severe pain at the end of his/her life is also a patient of no recovery. On the other hand, with regard to the goodness of fit index, it was shown that the three models have had a great fit although the best fit of the model could be the third one. The factorial solution of this third model has identified similar factors with the original: severe pain, no recovery, patient request and doctor’s authority. However, the main difference was that several items did not have factorial weights in several factors. For instance, items 1 and 3 where in the original instrument could define the factor severe pain or patient request. On the contrary, in the model two and three these items were just in a factor: severe pain. In this study, we tried to identify the dimensions of ATE and checked the best factorial structure and best fit model according to the number of factors. In this sense, it seems more appropriate that although it is possible to identify the 4 dimensions, since the items that make up the test are so few, and although they are indeed dimensions that can influence a response or a more favorable attitude towards euthanasia, the test should be evaluated from a single dimension as a result of all the items in terms of the attitude of these professionals towards euthanasia.

The original scale includes also a conceptual continuum between active or passive euthanasia, which we have not been able to demonstrate statistically since at no time have, we identified a two-dimensional model that includes these items. However, we do find it very interesting that the author identifies this difference through the different items because in reality it would be very interesting to be able to determine the different attitudes towards passive or active euthanasia, taking into account that these two terms will generate different attitudes. According to the study of Young Ho Yun et al. in 2018 [22] physicians had a more negative attitude toward active ending of life than the other groups (including patients with cancer, family caregivers and general population). Moreover, in the same study women were less likely than men to prefer passive euthanasia. In a study carried out recently in Mexico with 1300 medical students, 44% were in favor of active euthanasia while 56% of passive euthanasia [23].

Nurses clearly play an important role in providing the necessary end-of-life care 24 h a day, not only in symptom control and pain management so important in certain terminal illnesses, but also in the emotional support they provide to the dying patient and their family [24]. It is therefore not surprising that they are the first to receive the first demand for euthanasia and therefore to be aware of the importance of getting involved in the current debate about euthanasia [25]. The attitude of nurses towards euthanasia has been well researched in countries where euthanasia is legal and even in countries where the nurse is beginning to play a crucial role in the final euthanasia assessment process or even, as has recently been the case in Canada, in carrying it out [26,27].

There have been some limitations in this study. Firstly, the sample was selected through non- probability sampling, which could introduce some bias into the results. However, the sample of health workers from several Spanish hospitals was enough large to apply the factorial analysis. Secondly, the scale is a self-report tool this have to be taken in account as the responder can biased responses.

## 5. Conclusions

To sum up, from the results of this study we can say that ATE is an instrument that can be use to measure attitudes towards Euthanasia in Spanish health workers. However, it would be necessary to replicate the study with several samples in order to reinforce the findings, specially about the number of factors identified and analysis the statistical results of several items of the scale.

## Figures and Tables

**Table 1:** Means, standard deviations, item homogeneity and α if item deleted for the ten items of the Attitude Toward Euthanasia Scale.

	M	SD	Skewness	Kurtosis	Item Homogeneity	α If Item Deleted
ATE 1	1.99	1.27	0.057	−1.07	0.506	0.813
ATE 2	1.75	1.24	0.194	−0.932	0.685	0.793
ATE 3	1.86	1.24	0.065	−1.07	0.626	0.799
ATE 4	1.97	1.17	−0.044	−0.890	0.604	0.802
ATE 5	1.80	1.14	0.173	−0.795	0.628	0.800
ATE 6	1.95	1.17	0.116	−0.886	0.305	0.832
ATE 7	1.57	1.07	0.304	−0.684	0.580	0.806
ATE 8	2.47	1.18	−0.513	−0.630	0.536	0.809
ATE 9	2.00	1.16	−0.010	−0.981	0.205	0.841
ATE 10	2.66	1.13	−0.649	−0.458	0.485	0.814

Standard kurtosis error = 0.341; standard skewness error 0.172.

**Table 2:** Factorial solution for the M1, M2 and M3.

	M_1_	M_2_			M_3_			
	1	1	2	3	1	2	3	4
ATE 1	0.626	-	-	0.885	-	-	0.875	-
ATE 2	0.790	0.711	-	-	0.711	-	-	-
ATE 3	0.743	-	-	0.790	-	-	0.778	-
ATE 4	0.714	0.841	-	-	0.810	-	-	-
ATE 5	0.743	0.764	-	-	0.755	-	-	-
ATE 6	0.365	-	-	-	-	-	-	0.820
ATE 7	0.711	0.704	-	-	0.723	-	-	-
ATE 8	0.656	-	0.898	-	-	0.842	-	-
ATE 9	-	-	-	-	-	-	-	0.749
ATE 10	0.599	-	0.844	-	-	0.897	-	-
% variance explained	35.45	20.82	25.40	6.38	20.85	25.99	5.91	4.55

* factorial weight less than 0.30 were removed.

**Table 3:** Fit indices for the ATE from Confirmatory Factor Analysis.

	x^2^	df	*p* Values	AGFI	CFI	NFI	TLI	RMR	RMSEA	AIC	BIC
M_1_	200.61	35	0.004	0.907	0.964	0.924	0.948	0.064	0.063	240.16	306.68
M_2_	19.61	13	0.005	0.907	0.964	0.928	0.942	0.060	0.067	100.10	162.86
M_3_	31.54	25	0.172	0.909	0.973	0.953	0.950	0.050	0.077	91.54	190.64
M_4_	69.60	29	0.000	0.883	0.942	0.943	0.909	0.075	0.084	121.60	207.49

Note: M_1_: unidimensional solution; M_2_: three factors according to Waserman et al. 2005; M_3_: four factors according to Waserman et al. 2005; M_4_: original scele; AGFI: Adjusted goodness of fit index; CFI: Comparative fit index; NFI: Normed fit index; Tucker-Lewis coefficient; RMR: Root mean square residual; RMSEA: Rood mean square error of approximation; AIC: Akaike Information Criterion and BIC: Bayesian Information Criterion.

**Table 4:** Comparative analysis of dimensions’ number between this model and Waserman et al.

Dimensions	Waserman et al. 2005	M1	M2	M3
Severe pain	1, 3, 9 (5–7)	One factor	1,3	1,3
No recovery	4, 6 (2, 8, 10)		-	6,9
Patient request	8, 10 (1,3)		8,10	8,10
Doctor’s Authority	4, 5, 7 (2)		2, 4, 5, 7	2, 4, 5, 7
